# Mechanisms of Immunotherapy Resistance in Cutaneous Melanoma: Recognizing a Shapeshifter

**DOI:** 10.3389/fonc.2022.880876

**Published:** 2022-04-19

**Authors:** Jessica Thornton, Gagan Chhabra, Chandra K. Singh, Glorimar Guzmán-Pérez, Carl A. Shirley, Nihal Ahmad

**Affiliations:** ^1^ Department of Dermatology, University of Wisconsin, Madison, WI, United States; ^2^ William S. Middleton Memorial Veterans Hospital, Madison, WI, United States

**Keywords:** immunotherapy resistance, melanoma, immune checkpoint inhibitors, immune signaling, overcoming immunotherapy resistance

## Abstract

Melanoma is one of the seven most common cancers in the United States, and its incidence is still increasing. Since 2011, developments in targeted therapies and immunotherapies have been essential for significantly improving overall survival rates. Prior to the advent of targeted and immunotherapies, metastatic melanoma was considered a death sentence, with less than 5% of patients surviving more than 5 years. With the implementation of immunotherapies, approximately half of patients with metastatic melanoma now survive more than 5 years. Unfortunately, this also means that half of the patients with melanoma do not respond to current therapies and live less than 5 years after diagnosis. One major factor that contributes to lower response in this population is acquired or primary resistance to immunotherapies *via* tumor immune evasion. To improve the overall survival of melanoma patients new treatment strategies must be designed to minimize the risk of acquired resistance and overcome existing primary resistance. In recent years, many advances have been made in identifying and understanding the pathways that contribute to tumor immune evasion throughout the course of immunotherapy treatment. In addition, results from clinical trials focusing on treating patients with immunotherapy-resistant melanoma have reported some initial findings. In this review, we summarize important mechanisms that drive resistance to immunotherapies in patients with cutaneous melanoma. We have focused on tumor intrinsic characteristics of resistance, altered immune function, and systemic factors that contribute to immunotherapy resistance in melanoma. Exploring these pathways will hopefully yield novel strategies to prevent acquired resistance and overcome existing resistance to immunotherapy treatment in patients with cutaneous melanoma.

## Introduction

Melanoma is one of the deadliest cancers of skin and its incidence has increased significantly at a constant pace in the past few decades in the United States. Historically, the diagnosis of distant metastatic melanoma has been associated with significant mortality. Before the invention of immunotherapies, less than 10% of patients who received such a diagnosis survived more than 5 years. From the 1990’s through 2010’s novel treatments were lacking for melanoma, until the approval of the first immune checkpoint inhibitor (ICI) in 2011 [reviewed in (1, 2)]. This discovery launched a revolution that resulted in this once death sentence into a better manageable disease with a 5-year survival rate as high as 44% [reviewed in (3)]. Melanocytes have complex interplay with the immune system, as spontaneous regression of nevi is common in early life, suggesting an active immune surveillance system that eliminates transformed melanocytes to prevent tumor formation (4, 5). However, neoplastic cells skew the immune system to an immunosuppressed state and acquire mechanisms to escape immune responses (6). This is reflected by a long lag between enhanced melanocytic proliferation to neoplastic progression (7).

Immune checkpoint inhibitors are drugs that block proteins known as immune checkpoints, which monitor the immune system (e.g. programmed cell death protein 1/programmed death ligand 1 [PD-1/PD-L1) and cytotoxic T-lymphocyte-associated protein 4 (CTLA-4)] (4). These checkpoints negatively regulate the immune response that normally protects the individual from severe reactions (8). In cancer patients, this prevents immune cells from targeting tumor cells, permitting malignant cells to evade the immune response, which is considered a hallmark of cancer (3). Presently, immune checkpoint inhibitors are some of the standard treatments used for melanoma patients.

The clinical trial data showing superior anti-melanoma effects of ICIs such as pembrolizumab (anti-PD-1), nivolumab (anti-PD-L1), and ipilimumab (anti-CTLA-4), over traditional therapies, has led to their rapid FDA approval (9, 10). However, approximately 66% of melanoma patients still experience disease progression while on immunotherapy and approximately 50% still die of melanoma (3). Thus, despite the promising clinical outcomes, immune checkpoint inhibitors have been associated with lower than desirable responses due to resistance leading to disease progression or relapse. The etiology of the resistance to immunotherapies that often underlies these deaths is often multifactorial. Mechanisms of immunotherapy resistance are poorly understood across all cancer types, and melanoma is no exception. Since the driving cause of resistance is not fully understood, it is currently not possible to identify which patients are likely to respond and which patients will develop resistance to a chosen immunotherapy before initiating treatment.

Although, there are various reviews available focusing on melanoma and immunotherapies, the goal of this review is to discuss the recent advancements towards elucidating the mechanisms of melanoma resistance to immunotherapies. As this is a pressing area of research, the focus will be on tumor intrinsic characteristics of resistance, epigenetic mechanisms of resistance, altered immune function, and systemic factors that contribute to immunotherapy resistance. Due to the complexity of these pathways, there is some overlap between the groups. We have also discussed the possible ways to overcome the resistance to immunotherapies in melanoma.

## Current Immunotherapies for Melanoma

### Immune Checkpoint Inhibitors (ICIs)

A number of ICIs are being currently used for melanoma treatment. Ipilimumab is an anti-CTLA-4 monoclonal antibody, meaning that it binds to CTLA-4 protein, blocking the interaction between CTLA-4 and its ligands (CD80 and CD86), and consequently inhibiting the activity of this protein (11, 12). This negative regulation activates the proliferation of T cells and promotes the attack of cancerous cells, including melanoma (13). Ipilimumab was one of the first treatments to prolong the survival of metastatic melanoma patients and indeed, after FDA approval in the year 2011, National Comprehensive Cancer Network recommended it as category 1 for patients with late-stage melanoma (12, 14). Ipilimumab is recommended for unresectable or metastatic melanoma cases, and as an adjuvant therapy. This marked a milestone in immunotherapy, as selected patients reaped long-term responses after treatment. Survival increased in some for up to 10 years (15). On the other hand, at the beginning of treatment, some patients experience atypical responses or pseudo-progression —meaning that they experience an increase in tumor size and/or new lesions present due to a weak antitumor immune response, or even immune effector cells aiding tumor growth (16). However, continued treatment results in an anti-melanoma response (16). Moreover, since ipilimumab stimulates T-cell proliferation, it can lead to immune-related adverse effects (irAEs) like dermatitis, endocrinopathy, and hepatitis, and other side effects including pruritus, fatigue, and colitis, the latter being the most prevalent (12, 14, 15). Fortunately, most of these reactions can be treated and even reversed. Even though a higher percentage of patients (20-25%) experience long-term beneficial effects including increased survival rates, overall response rates range on the lower end (12-19%) (13). Therefore, researchers have been searching for prognostic and predictive biomarkers that could help determine which patients would benefit from this treatment the most (12). Interestingly, patients who experience changes in blood markers during treatments, like increased counts of eosinophil and lymphocytes, have better outcomes (17). Nonetheless, more biomarkers are being explored including peripheral blood cell markers, molecular markers, and even markers found in the gut microbiome (17, 18). Overall, due to the low efficacy and high cost (approximately $80,000-$120,000 per patient) other treatments are now preferred.

In 2014, pembrolizumab (anti-PD-1) and nivolumab (anti-PD-L1) were approved as melanoma treatments and are currently used as first-line therapy for patients with advanced melanoma (1, 8, 19). Both of these drugs work by inhibiting PD-1, an immune checkpoint that, unlike CTLA-4, is activated later in the immune response since it is expressed after continuous exposure to antigens (1). PD-1 is expressed in T cells and after biding to its ligands (PD-L1 and PD-L2) decreases the proliferation, activity, and survival of T cells (11, 20). Interestingly, cancer cells often increase the expression of PD-L1, which allows them to evade being targeted by immune cells (11, 20). Therefore, inhibiting PD-1 stimulates immune cells to attack tumor cells (20). Nivolumab is used as monotherapy, combination therapy, or adjuvant therapy to treat patients with unresectable or metastatic melanoma (20). Monotherapy with this agent was shown to increase survival and response rate (up to 44%) in advanced melanoma patients (15). Some patients experience minor and manageable side effects (e.g. diarrhea, nausea, pruritus) and/or irAEs, but nivolumab is considered safe and effective (21). Comparatively, pembrolizumab imparts long-term antitumor effects (more than 5 years), increases survival in advanced melanoma patients, and decreases the risk of disease progression and death in more than one-third of the patients (22–24). Interestingly, treatment regimens are adaptable, but the efficacy remains constant and depending on the patient, treatment can go on for up to two years (16, 24). Likewise, this immunotherapy agent is well-tolerated by melanoma patients since the adverse effects are less frequent, thyroid disorders being one of the most frequent effects observed (22, 23). Treatments with nivolumab and pembrolizumab have been more effective than ipilimumab monotherapy, yielding increased survival rates in 35%-50% of patients as well as having a favorable toxicity profile and lower rates of irAEs (3, 25). Overall, checkpoint inhibitors are modern immunotherapeutics that have been shown to improve melanoma patient prognosis in resectable tumors as well as metastatic melanoma (13, 19).

### Oncolytic Enhanced Immunotherapy

In 2015, FDA approved the use of talimogene laherparepvec (T-VEC or Imlygic) for advanced melanoma patients, becoming the first oncolytic virus therapy (OVT) in the United States [reviewed in (26)]. In fact, OVT leads to oncolysis, apoptosis, necrosis, autophagic cell death, and stimulates an overall anti-tumor immune response (27). T-VEC is an oncolytic herpes simplex virus type 1 (HSV-1) that has mutations in several genes including c34.5, a47, and granulocyte-macrophage colony-stimulating factor (GM-CSF) (27). Particularly, the first two genes are deleted while the latter is a transgene derived from humans that is inserted into the deleted c34.5 loci of the virus [reviewed in (28)]. Deletion of c34.5 is crucial for the virus to selectively replicate in cancer cells without infecting normal cells, while a47 usually negatively regulates antigen presentation, hence its deletion promotes antitumor immune response (29, 30). On the other hand, the reasoning behind adding GM-CSF relies on its potential of enhancing antitumor activity, nevertheless, researchers have suggested that other human genes might be preferred (e.g. interleukins 12 and 18) due to their key immune-related roles [reviewed in (28)]. The immunosuppressive TME favoring melanoma ICI resistance can be taken advantage of as a target for viral attack; dysfunctional immune signaling allows genetically engineered non-pathogenic viruses to selectively target cancer cells and consequently replicate in them to a greater extent than in normal cells (28, 31). OVTs have been associated with activation of T and NK cells, release of immunogenicity stimulating agents, release of tumor-specific antigens for APC uptake, type I IFN signaling, and major histocompatibility complex (MHC) upregulation (32, 33). The overall response rate to T-VEC monotherapy is only around 25% (34), but the true promise of OVTs arguably lies in their immunomodulatory properties.

#### T-VEC and Anti-PD-1 Therapy

Tumor adenovirus OVT injection drastically alters the immune landscape with increased NK, T cell and APC migration occurring. Interestingly, initial trials observed that OVTs were associated with an increase of PD-1 and PD-L1 expression (32). In response to these findings, recent reports reveal PD-L1 expression is an adaptive mechanism used by melanoma to generate OVT resistance (33, 35). The exact relationship between PD-L1 expression and anti-PD1 efficacy remains cloudy, but multiple studies associate high PD-L1 with improved therapeutic outcomes (36, 37). This suggests OVTs may prevent anti-PD-1 resistance acquisition through PD-L1 regulation, or even reverse acquired resistance. This hypothesis is supported by a study that found Newcastle Disease Virus (NDV) sensitized B16-F10 tumors to anti-PD-1 therapy (33). T-VEC was also found to enhance anti-PD-1 therapy responses (38). Another recent study taking advantage of PD-L1 OVT response generated a novel OVT that expressed a PD-L1 inhibitor. This “double-armed” OVT reactivated T cell responses and specifically targeted B16-F10 melanoma alone, or even more effectively when also paired with PD-L1 antibodies (39). The re-activation of tumor immune pathways in response to viral infection already gives a basis for sensitization to ICIs, but the possibility to overcome resistance problems through OVT mediated PD-L1 control provides an appealing prospect for further research.

#### T-VEC and Anti-CTLA-4 Therapy

T-VEC also positively augments anti-CTLA-4 therapies in melanoma, as do other OVTs (38, 40). A measles OVT was developed to express CTLA-4 antibodies and showed promising results in human melanoma xenografts (41). A recent study using an anti-CTLA-4 expressing NDV OVT found the treatment was similar in effectiveness to traditional anti-CTLA-4 therapy in combination with radiation, in B16-F10 melanoma (42). Zamarin et al. have discussed how NDV treatment reactivates the melanoma immune landscape through T and NK cells, MCH I and II, and interferon signaling to provide a synergistic effect when combined with anti-CTLA-4 therapy in B16-F10. They also found NDV therapy increased the expression of CTLA-4 ligands (CD80 and CD86), thus providing a potential mechanism for direct modulation of CTLA4 resistance (43). The interplay between OVTs and anti-CTLA-4 resistance is poorly researched, but initial studies show promising results for both combined therapies and insight into potential mechanisms for ICI therapy resistance deterrence *via* OVTs.

#### T-VEC and STING Signaling

As a master regulator of innate immunity pathways, it is unsurprising that STING signaling inhibits OVT efficacy (31, 44, 45). A recent study used T-VEC to effectively target STING deficient melanoma (31). OVTs have good potential for targeting ICI-resistant tumors as the same pathways developing resistance also develop viral susceptibility. Furthermore, if STING reactivation occurs as a result of OVT, it may re-sensitize melanoma to ICIs. This remains untested but should be investigated as it may provide a basis for therapies alternating between OVT and ICI therapy admission. Overall, OVTs directly augment ICI therapy efficacy through their viral-induced immune responses. Many OVTs have poor lytic capabilities, but recent combinations with ICI therapies have revealed potential possibilities for avoiding secondary resistance or overcoming it in primary instances. The relationship between OVTs and known resistance-pathways desperately need to be studied so that novel, exploitive, and highly effective OVT/ICI combinations can be employed.

## Key Players in Resistance Against Immune Checkpoint Inhibitors (ICI)

Despite promising clinical results, immune checkpoint inhibitors have been associated with sub-optimal responses to single-agent therapies due to resistance or relapse. Around half of advanced melanoma patients develop resistance to PD-1 treatments [reviewed in (25)]. Even though antitumor effects from anti-PD-1 and anti-CTLA-4 therapies are durable, they are associated with low response rates likely due to intrinsic and acquired resistance to treatments [reviewed in (8, 46)]. For these reasons, researchers have been exploring the potential of combining treatments (multiple immunotherapies as well as other types of treatments together with immunotherapies). This pertains especially to nivolumab and pembrolizumab since they are well tolerated by patients and have the most promising outcomes.

**Figure 1:**
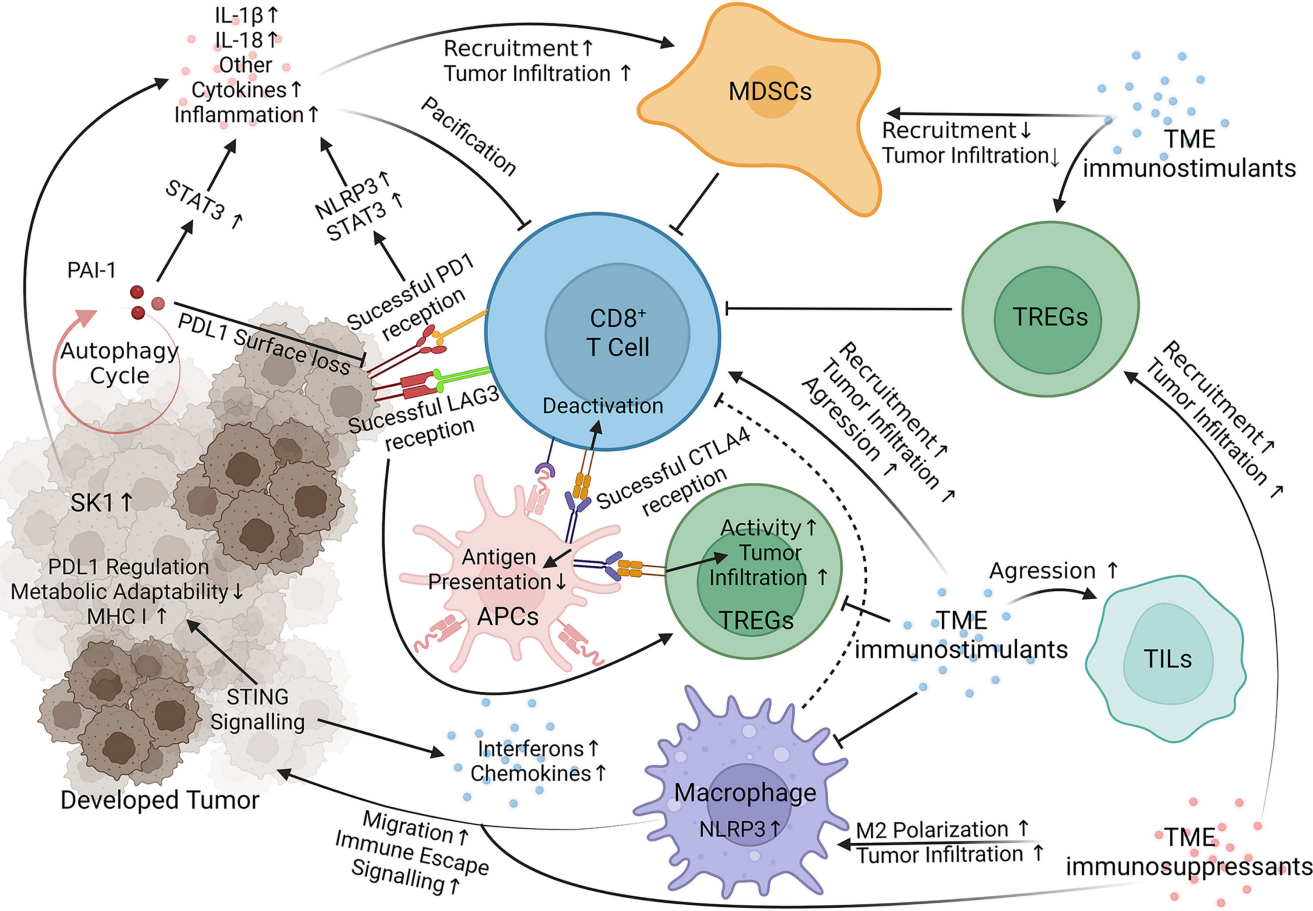
Immunotherapy Resistance Signaling. Key targets of interest negate therapeutic responses through internal melanoma pathways, acquisition of TME `immunosuppressants (red) and loss of TME immunostimulants (blue). STING, Stimulator of Interferon Genes; NLRP3, Nucleotide-binding domain, leucine-rich containing family, pyrin domain-containing-3; PAI-1, Plasminogen Activator Inhibitor-1; LAG3 (Green on T Cell), Lymphocyte-activation gene 3; SK-1, Sphingosine kinase 1; PD1, Programmed cell death protein 1 (yellow on T Cell); CLTA4, Cytotoxic T-Lymphocyte Associated Protein 4 (Blue on APC); STAT3, Signal transducer and activator of transcription 3; IL, interleukin; MDSCs, Myeloid-derived suppressor cells; TREGs, Regulatory T cells; TILs, Tumor-infiltrating lymphocytes; APCs, Antigen Presenting Cells (45, 56–78). Visualization created with BioRender.com.

Beyond honing the current therapeutic arsenal, melanoma targets governing resistance are also under investigation. T cell immunoglobulin and mucin domain 3 (TIM-3) and T cell immunoreceptor with Ig and ITIM domains (TIGIT) are two extensively studied immune checkpoints responsible for fortifying melanoma against current immunotherapies, especially anti-PD-1 treatments (47) and [reviewed in (48–51)]. The human leukocyte antigen (HLA) complexes I and II are responsible for antigen presentation, a crucial factor in lymphocyte function. HLA malfunction in melanoma is well documented and responsible for deterring anti-PD-1 and anti-CLTA-4 consequences [reviewed in (8, 52)]. The tumor microenvironment (TME) is another well-reviewed emerging area of interest in immunotherapy resistance research [reviewed in (53, 54)]. Indoleamine 2,3 dioxygenase-1 (IDO) is another target shown to manifest immunotherapy resistance through the accumulation of TME immunosuppressants (55) and reviewed in (50). Past these well-documented resistance factors, newer reports detail many other mechanisms utilized by melanoma to impede immunotherapies. Below, we summarize the most recent advancements towards elucidating the signaling mechanisms of melanoma resistance to immunotherapies (**Figure 1** and **Table 1**).

**Table 1:** Recently revealed drivers of immunotherapy resistance and their influences.

Drivers of Immunotherapy Resistance	Immune Influence(s)	References
CpG promoter	Methylation of melanoma genes of interest	(79)
EZH2	MHC I expression, antigen presentation, CD8^+^ T cell infiltration, STING regulator	(56)
HDAC6	IL-10 and PD-L1 expression	(80)
RASSF5 and ITGB2	Immunogenicity Generation	(80)
KDM5B	Recruits the H3K9 methyltransferase SETDB1	(81)
SETDB1	Regulates expression of immune-related gene clusters, MHC I expression, antigen presentation	(82–84)
FTO	Increased PD-1 expression through autophagy	(85)
TMB	Generates neoantigens to promote successful immunosurveillance	(8, 86–88)
LAG3	APC activator when bound to MHC II and negative regulation of T cells	(89–91)
SK1	Lymphocyte trafficking and differentiation	(92, 93)
STING	MHC I expression, metabolic regulation, PD-L1 expression, immunostimulating interferon and chemokine signals	(45, 57–61)
NLRP3	Inflammatory interleukin signals, MDSC control, macrophage polarization	(64–69)
PAI-1	Macrophage polarization, PD-L1 surface levels, autophagy cycle, TREG control	(70–73)
Microbiome	Activation of various pathways (including STING) involved in T cell response and interleukin signaling	(94–99)
VEGF	Inhibition of dendritic cell maturation and T cell tumor infiltration	(100, 101)

### Epigenetic Mechanisms of Resistance

Epigenetics is a rapidly evolving field that studies changes in gene expression that occur without modification of the underlying DNA sequence. Major epigenetic processes often involve the addition or removal of compounds to either DNA sequence itself, or the histones DNA is wrapped around. Histone acetylation, deacetylation, and methylation are often carried out by histone acetyltransferases (HATs), histone deacetylases (HDACs), and histone methyltransferases (HMTs), respectively. Similar impermanent modifications can also be made to regulatory elements in DNA by a variety of enzymes, including DNA methyltransferases (DNMT). Epigenetics is intertwined with melanoma resistance to immunotherapies through the modulation of proteins that promote resistance. Aleotti et al. authored a review article that covers many of the key roles of global loss of DNA methylation with promoter CpG island hypermethylation for tumor promotion and lists many hypermethylated and hypomethylated genes that are associated with melanoma (79).

A review by Gracia-Hernandez et al. has described many of the epigenetic modifiers involved in melanoma pathogenesis, resistance to targeted and immunotherapies, and potential epigenetic targets for therapeutic adjuvants. They discuss the role of reduced 4-1BB (CD137) expression by DNA hypermethylation, HDAC6 promotion of IL-10 and PD-L1 expression, and mutations in the histone methyltransferase EZH2, which reduces expression of proteins involved in the antitumor immune response, such as RASSF5 and ITGB2 [reviewed in (80)]. Recent research has expanded the association of EZH2 with immune evasion. Xu et al. revealed that EZH2 inhibition enhances STING expression and signaling. Combining EZH2 inhibition with STING agonist successfully suppressed melanoma in a mouse model (B16-F10) with poor immunogenicity. These mice displayed increased MHC I expression and antigen presentation, increased CD8^+^ T cell infiltration of tumors, and improved survival (56). This finding is promising for the development of a therapy to combat immune evasion in melanoma that has demonstrated resistance to immunotherapies.

Recent research has identified several epigenetic regulators that are altered in melanoma and may contribute to immunotherapy resistance. KDM5B is a H3K4 demethylase that has been implicated in melanoma resistance to targeted BRAF inhibitor therapies (102). However, recent studies have shown that KDM5B also promotes immune evasion in mouse models of melanoma by recruiting a H3K9 methyltransferase, SETDB1 (81). SETDB1 is enriched in many human cancers and its overexpression is associated with suppressed anti-tumor immune activity and resistance to immune checkpoint blockade (82–84). SETDB1 suppresses the anti-tumor immune response by blocking the expression of immune-related gene clusters and transposable elements that encode MHC I antigens recognized by T cells (82). The use of mithramycin A and mithramycin analog EC-8042 in melanoma cells enhanced the efficacy of MAPK inhibitor therapies (83, 103). However, despite the clear impact of KDM5B and SETDB1 on immune evasion, there have not been any studies on the effects of their inhibition in melanoma resistance to immunotherapies. The results of the above studies on KDM5B and SETDB1 indicate that an epigenetic axis is likely involved in tumor immune evasion, as discussed by Galassi et al. (104). Further research into this epigenetic regulator axis may help elucidate therapeutic targets for melanoma that have successfully evaded immune surveillance with current immunotherapies.

Fat mass- and obesity-associated (FTO) protein is a m6A RNA demethylase that is strongly linked to obesity and is highly expressed in certain types of acute myeloid leukemia (105). In melanoma, overexpression of FTO is associated with increased growth, proliferation, cell migration, and invasion (85). It is upregulated when melanoma cells are placed under conditions of metabolic stress, through activation of the autophagy pathway that also increases PD-1 expression (85). In a study using immunocompetent C57BL/6 mice with B10-F10 melanoma tumors as a model for melanoma resistance to immunotherapies, FTO knockdown restored the efficacy of anti-PD-1 blockade and significantly inhibited tumor growth (85). Another recent study showed that Dac51-mediated inhibition of FTO reduced the growth of injected B16-OVA melanoma tumors (106). A combination of Dac51 and anti-PD-1 checkpoint blockade demonstrated even slower tumor growth and prolonged survival of mice without evidence of significant general toxicity (106). The results are promising for the use of FTO inhibition as an adjuvant to current ICI approaches. Further research to identify selective FTO inhibitors and more research on their effects in melanoma models are necessary before these results can be adapted to human trials.

### Tumor Mutational Burden (TMB) and Neoantigens

A major factor that impacts the immunogenicity of cancers is tumor mutational burden (TMB), or the number of nonsynonymous mutations present in a tumor. Higher TMB indicates a greater number of mutations, which in turn increases the likelihood that an abnormal protein may be identified by the immune system. These proteins are termed neoantigens. Consistent with this theory, high TMB is generally associated with improved overall survival and immunotherapy response in melanoma patients (8, 86–88, 107). However, there is still some debate about a good way to make high and low TMB definitions more consistent across studies to improve its use as a biomarker for immunotherapy response [reviewed in (108, 109)]. Recent research has shown that TMB and neoantigen burden scores can predict response and likelihood of resistance to immunotherapies (110). However, these scores are limited when mutations that affect antigen presentation are present, which is true in approximately 50% of melanoma tumors (8). Many of the mechanisms underlying this loss are discussed in a recent review by Olbryt, Rajczykowski, and Widlak (8). In addition, immunotherapy creates positive selective pressure for low TMB cells. Patients demonstrating melanoma progression post-ipilimumab treatment subsequently treated with nivolumab saw a decreased TMB when this course of action was effective (111). If a given tumor has subclones with different levels of neoantigen burden, the ICI therapy may eliminate all high TMB clones but leaving low TMB clones behind, resulting in a newly resistant tumor (111). Research to develop combination therapy that targets both hot and cold immunogenic subclones may decrease rates of acquired resistance by preventing this selection.

### Altered Immune Signaling

#### Lymphocyte-Activation Gene 3 (LAG3)

Lymphocyte-activation gene 3 (LAG3) is both an activator of antigen-presenting cells when bound to MHC II and a negative regulator of T cell activation when found on T cells. Because of this mechanism, LAG3 has both been associated with non-response to therapy and therapeutics mimicking it has been used to induce antitumor immunity (52, 89–91). Elevated serum concentrations of soluble LAG3 are seen in non-responders to anti-PD1 therapy and increased tumor infiltration with T cells positive for LAG3 and TIM3 was also correlated with shorter progression-free survival in patients receiving anti-PD1 immunotherapy (52, 90). To corroborate these findings, preliminary results from a recent clinical trial combining a monoclonal antibody against LAG3 (relatlimab) with nivolumab showed improved progression-free survival in patients treated with both relatlimab and nivolumab compared to only nivolumab (112). To note, the cohort treated with the combination relatlimab and nivolumab treatment did have a higher rate of treatment-related adverse events than the cohort only treated with nivolumab (112). More analyses regarding overall survival and objective response are forthcoming, but the initial evidence supports the theory that LAG3 may play a role in impairing response to immunotherapies. Moreover, a clinical trial combining eftilagimod alpha, a soluble LAG3 protein that activates antigen-presenting cells, with pembrolizumab, also shows promising activation of antitumor immune activity (89). Further research focusing on the many roles of LAG3 in driving antitumor immunity is necessary to determine the optimum therapy that can hijack this pathway to overcome and prevent resistance to immunotherapies.

#### Sphingosine Kinase 1

Sphingosine kinase 1 (SK1) is a kinase that catalyzes the phosphorylation of sphingosine to sphingosine-1-phosphate (S1P), an important regulatory protein for lymphocyte trafficking and differentiation (92, 93). Past research has shown that SKI is overexpressed in melanoma and causes elevated levels of S1P, though the mechanism through which this impacts immunotherapy response is still unknown (93). However, high expression of SKI in melanoma cells has been shown to be associated with resistance to anti-PD-1 immunotherapies in patients (93). In accordance with this, inhibition of SKI has been associated with the enhancement of the effect of anti-CTLA-4 and anti-PD-1 immunotherapies on melanoma cells *in vitro* (93). This is promising as several therapies that target the S1P axis have been approved by the food and drug administration for use in conditions like multiple sclerosis, and several S1P modulators are under investigation for use in various human cancers (92). Further preclinical and clinical research is necessary before these agents are ready for adaptation to melanoma treatment, but the initial preclinical data appears promising.

##### Stimulator of Interferon Genes Protein (STING)

Stimulator of interferon genes (STING) is an endoplasmic reticulum (ER) protein that regulates the immune response through interferon (IFN) and chemokine signaling (45, 57–61). STING signaling is often suppressed or even absent in melanoma (45). Activation of STING signaling was recently shown to increase MHC I expression and promote the T cell response against melanoma through increased type I IFN, and chemokine CXCL10 activity (57). In B16-F10 mouse models, STING stimulation improved anti-CTLA-4 and anti-PD-1 therapy outcomes through IFN signaling (62). The small molecule agonist of STING, diABZI, was shown to prevent Nuclear Factor Erythroid 2–Related Factor 2 (NRF2) activation in melanoma (58). NRF2 is relevant to therapy resistance as it regulates PD-L1 expression and drives oxidative metabolic adaptation (58, 63). Another recent study modified DNA methylation to enhance STING expression; this approach vastly increased TIL-mediated killing in melanoma (57). As discussed previously, EZH2 inhibitor mediated acetylation enhanced STING expression to improve therapeutic effects. STING agonist-loaded lipid nanoparticles (STING-LNPs) are a third method currently under investigation to enhance STING signaling. STING-LNPs diminished anti-PD-1 resistance in B16-F10 metastatic melanoma *via* IFN signaling, and synergistically provided an antitumor effect (60). Countering the suppression of STING signaling is an emerging method to improve the innate anti-tumor response and immunotherapy effectiveness while deterring therapy resistance. Clinical trials promoting STING signaling should be developed, and their results studied to further understand how these potent effects are elicited.

##### Nucleotide-Binding Domain, Leucine-Rich Containing Family, Pyrin Domain-Containing-3 (NLRP3)

NLRP3 regulates the caspase 1-dependent release of proinflammatory interleukins IL-1β and IL-18 through the formation of the NLRP3 inflammasome (64, 65). IL-1β is a known pro-tumorigenic and immunosuppressive agent in melanoma (66, 67). The orally active inhibitor, OLT1177, was recently used to target NLRP3 in a B16F10 mouse model. This approach resulted in decreased progression, inflammation, and normalization of Myeloid-Derived Suppressor Cell (MDSC) levels. OLT1177 combined with anti-PD-1 therapy further decreased MDSC levels and promoted T Cell tumor infiltration for an enhanced antitumor effect (67). These results were consistent with another recent study that both silenced NLRP3, or inhibited it with the small molecule MCC950, to report a greatly reduced MDSC presence and increased anti-PD-1 therapy response. NLRP3 activity is especially pertinent in the scope of immunotherapy, as MDSC recruitment was recently identified as a driver of resistance acquisition. Furthermore, this recruitment is unavoidable as PD-L1 was found to inhibit STAT3, which in turn activates the NLRP3 inflammasome (68). However, the role of NLRP3 in melanoma is unclear as another recent report found its activity induces cell death in BRAF inhibitor-resistant melanomas and improves prognosis (69). The results of NLRP3 manipulation may therefore depend on unknown genetic primings previously introduced through therapy. NLRP3 is also active in the TME; NLRP3 inhibition in macrophages with Celastrol resulted in decreased B16-F10 migration (66). The role of NLRP3 in negating immunotherapy resistance should be further explored, especially as a key component in adaptive anti-PD-1 resistance.

#### Plasminogen Activator Inhibitor-1 (PAI-1)

Plasminogen activator inhibitor-1 (PAI-1) is a member of the serine protease inhibitor family, specifically responsible for inhibition of tissue plasminogen activator and urokinase (70–73). PAI-1 was previously reported to promote macrophage M2 polarization and tumor infiltration through an IL-6/STAT3 pathway (72, 74). PAI-1 inhibition with tiplaxtinin in a B16-F10 mouse model decreased M2 macrophage and TREG tumor infiltration (73). Exogeneous PAI-1 was recently shown to also internalize PD-L1 in B16-F10 melanoma through endocytosis mediation. In a B16-F10 mouse model, PAI-I inhibition with tiplaxtinin prevented PD-L1 surface loss and provided a synergistic anti-tumor effect when combined with anti-PD-L1 therapy (70). Interestingly, a separate study also verified authophagically derived exogenous PAI-1 contributes to an immunosuppressive TME in melanoma mouse models. Melanoma cells challenged with mitoxantrone used autophagic PAI-1 release to gain resistance, suggesting a similar mechanism may be employed in immunotherapy cases (72). PAI-1 is also a known regulator of autophagy and its ability to create a paracrine positive feedback loop fostering further secretion has been verified, although not in melanoma (72, 73). Targeting PAI-1 may prove useful to deter treatment resistance through negation of autophagic signaling, macrophage polarization and halting of PD-L1 loss. The exogenous nature of PAI-I also merits further investigation in the scope of novel treatment development focused on TME modification.

## Overcoming Immunotherapy Resistance

The immune system plays a key role in cancer pathogenesis, prognosis and therapy responses. The use of ICI in cancer immunotherapy aims to target the interaction between immune cells and cancer cells, enhancing the immune system’s capabilities against tumors. Melanoma has been characterized as one of the most immunogenic tumors due to the existence of tumor-infiltrating lymphocytes (TILs) in resected melanoma and positive clinical responses to immune stimulation (113). Due to high immunogenicity, melanomas have had widespread success in being treated using ICI. The major advantage to immunotherapy over targeted therapy is the more durable response on cancer growth that can be present even after the drugs have been discontinued. However, a large percentage of partial responders (primary resistance) and high rates of resistance acquisition remain the greatest obstacles to the optimal success of these therapies (114, 115). As discussed above, immunotherapy resistance is the result of developing multiple interactions between cancer cells and the immune system. Thus, it appears that the optimal treatment of melanoma is likely to involve therapeutic regimens that include multiple agents, given together or in sequence, with molecularly defined targets. Below, we have addressed some of the strategies that may synergize with ICIs for maximal anti-melanoma responses.

### Use of Molecularly Targeted Therapy in Combination With Immunotherapy

This approach relies on the combinations of drugs targeting two different signaling pathways to induce apoptosis leading to the release of tumor-associated antigens, and/or modulating key cellular pathways that allow cancer cells to maintain an adaptive resistance. BRAF inhibitors, such as dabrafenib and vemurafenib, have demonstrated a survival advantage as both monotherapy and in combination with MEK inhibitor trametinib in both resectable and unresectable or metastatic melanomas (116–118). However, most of the patients ultimately acquire resistance, thereby failing to achieve durable tumor regression (119). Interestingly, preclinical and clinical studies combining anti–PD-1/PD-L1 with BRAF/MEK inhibitors have demonstrated enhanced anti-melanoma responses and tolerability (120). Similarly, bevacizumab, an anti-vascular endothelial growth factor (VEGF) monoclonal antibody, has been found to possess immunomodulatory properties, as VEGF exerts immunosuppressive functions *via* inhibiting dendritic cell maturation and T-cell tumor infiltration. With this rationale, several clinical trials are evaluating the combination of immune checkpoint inhibitors with anti-VEGF therapies across multiple tumor types including melanoma (100, 101).

### Concurrent Inhibition of Two or More Immune Checkpoints

Combination therapy with anti–CTLA-4 and anti–PD-1 has been approved or in clinical trials for certain cancers including melanoma. In a phase I clinical study, involving 142 patients with metastatic melanoma, the objective-response rate and progression-free survival have been found significantly higher with ipilimumab (CTLA-4 inhibitor) and nivolumab (PD-1 inhibitor) combined therapy than ipilimumab monotherapy (121). Three years post-trial, the average survival rate of the nivolumab-plus-ipilimumab group was 58%, compared to 52% for nivolumab alone (122). At five years, the survival rate was 52% for nivolumab-plus-ipilimumab and 44% for nivolumab alone (123). At six and a half years, the survival rate was 49% for nivolumab-plus-ipilimumab and 42% for nivolumab alone (124). These results demonstrate the durability of the response achieved through utilizing nivolumab alongside ipilimumab (124). The clinical benefits with this combination therapy may have been due to complementary mechanisms as ipilimumab is known to prime T cells, whereas nivolumab reactivates effector responses. Similarly, anti-PD-L1 along with anti-CTLA-4 and radiotherapy has been demonstrated to promote a better response in a subset of patients with metastatic melanoma (125).

### Influence of the Microbiome

Several studies have noted the connection between gut microbiome and melanoma response to immunotherapy, and postulated some theories for how microbiome influences immunotherapy responses (94–97). In patients with microbiota that favors immunotherapy response, microbiota-derived STING agonists induce IFN1 signaling and spur anti-tumor immune response (94, 95). It is theorized that gut microbiome may influence response to immunotherapy through the production of short-chain fatty acids and their subsequent influence on the epigenome of melanoma cells. A recent study found that pentanoate induced epigenetic reprogramming of T cells by inhibiting class 1 histone deacetylases, increasing mTOR activity in CD8^+^ T cells, and enhancing expression of CD25 and IL-2, which empirically increased anti-tumor activity of CD8^+^ T cells treated with these short-chain fatty acids (96). In one study, oral administration of probiotic *Bifidobacterium* in combination with PD-L1 has been found to almost abolish melanoma tumor growth in a mouse model (98). The molecular analyses suggest that the effects were mediated by improved dendritic cell function resulting in enhanced CD8^+^ T cell priming and accumulation in the TME (98). In an analysis of fecal microbiome samples of melanoma patients undergoing anti-PD-1 immunotherapy, diversity and composition of gut microbiome of responders showed significantly higher alpha diversity and relative abundance of bacteria of *Ruminococcaceae* family compared to nonresponders (99). Moreover, fecal microbiota transplantation has been found to overcome resistance to PD-1 blockade in germ-free mice (99). Based on these promising preclinical data, a small phase 1 clinical trial of fecal microbiota transplant (FMT) and reinduction of anti-PD-1 therapy has been conducted in patients with melanoma refractory to initial anti-PD-1 immunotherapy (97). The FMT was sourced from patients with melanoma who responded to anti-PD-1 treatment (97). The study found that FMT was associated with favorable changes in immune cell infiltrates and deemed FMT safe to pursue in larger phase clinical trials (97). If replicated in these larger trials, FMT could be a viable option for patients who experience melanoma progression while on anti-PD-1 immunotherapy.

### Other Combination Strategies With Immunotherapy

Similarly, several other combination therapeutic strategies to overcome immunotherapy resistance are being investigated [reviewed in (126)]. For example, combining cancer vaccines with ICI has been found to be beneficial in multiple preclinical studies as it increases antigen presentation and prime T cells. Enhanced survival has been noticed with multi-peptide vaccine and nivolumab adjuvant therapy in melanoma patients (127). In another study, a personalized neoantigen vaccine (that targets up to 20 predicted tumor neoantigens) paired with anti-PD-1 therapy showed complete tumor regression in melanoma patients (128).

Further, a small molecule IDO inhibitor Epacadostat was tested in combination with pembrolizumab in a metastatic melanoma trial with 928 patients, however, progression-free survival was not significantly affected (129). Other Phase 1 and 2 trials combined the nuclear factor erythroid 2-related factor 2 (NRF2) agonist, Omaveloxolone, with Ipilimumab or Nivolumab in hopes of abrogating MDSC-driven immunosuppression (ClinicalTrials.gov Identifier: NCT02259231). Omaveloxolone was associated with decreases in tumor iNOS, PD-L1, and IDO-1 expression without any dose-limiting toxicities and thus, it may overcome ICI resistance (130). Furthermore, Ipilimumab was also combined with oncolytic virus (T-VEC) therapy, yielding, a 50% progression-free survival and 67% overall survival at 18 months demonstrating superiority over T-VEC monotherapy. (ClinicalTrials.gov Identifier: NCT01740297) (131). Given these promising findings, several new clinical trials have been initiated to devise the strategies to overcome immunotherapy resistance in melanoma. Concurrently, research investigations are also underway to identify biomarkers associated with ICI resistance and treatment responses.

## Conclusion

Melanoma has been characterized as one of the most immunogenic tumors due to the existence of TILs in resected melanoma, occasional spontaneous regressions, and clinical responses to immune stimulation. The immunogenicity of melanoma has led researchers to identify novel immune strategies to overcome tumor immune evasion. Nevertheless, high rates of resistance acquisition, lack of long-lasting anti-melanoma responses and higher percentages of limited responders remain key obstacles to the realization of immunotherapies. The mechanisms of immunotherapy resistance need to be identified for successful future drugs targeting those mechanisms. There are definite advancements in current research exploring novel mechanisms of resistance against immunotherapy for melanoma. Since melanoma is notoriously resistant to treatment and current therapeutic approaches have not been able to effectively manage this neoplasm, there is no doubt that the future treatment of melanoma will involve therapeutic regimens that include multiple agents, given together or in sequence, with wide varieties of molecularly defined and immunologic targets.

## Author Contributions

Conceptualization, JT, GC, and NA. Writing—original draft preparation, JT, GC, CKS, GG-P, and CAS. Writing—review and editing, JT, GC, CKS, GG-P, CAS, and NA. Tables and figures, CAS, GC, and CKS. Supervision, NA. Project administration, NA. All authors contributed to the article and approved the submitted version.

## Funding

This work was partially supported by funding from the NIH (P30 CA014520) and the Department of Veterans Affairs (VA Merit Review Awards I01CX002210 and 1I01BX004221; and a Research Career Scientist Award IK6BX006041).

## Conflict of Interest

The authors declare that the research was conducted in the absence of any commercial or financial relationships that could be construed as a potential conflict of interest.

## Publisher’s Note

All claims expressed in this article are solely those of the authors and do not necessarily represent those of their affiliated organizations, or those of the publisher, the editors and the reviewers. Any product that may be evaluated in this article, or claim that may be made by its manufacturer, is not guaranteed or endorsed by the publisher.
